# Dosimetric Comparisons of Volumetric Modulated Arc Therapy and Tomotherapy for Early T-Stage Nasopharyngeal Carcinoma

**DOI:** 10.1155/2018/2653497

**Published:** 2018-06-04

**Authors:** Shan Li, Qin Zhou, Liang-Fang Shen, Huan Li, Zhan-Zhan Li, Zhen Yang, Ming-Jun Lei, Xiao-Yu Yang, Zi-Jian Zhang, Yong-Mei Hu, Ze-Fu Jin, Gui Liu, Zhi-Ping Lv, Xin-Qiong Huang

**Affiliations:** Department of Oncology, Xiangya Hospital, Central South University, No. 87, Xiangya Road, Changsha, Hunan Province 410008, China

## Abstract

**Purpose:**

To compare the dosimetric differences between volumetric modulated arc therapy (VMAT) and helical tomotherapy (HT) in treating early T-stage nasopharyngeal carcinoma (NPC).

**Method:**

Ten patients with early T-stage NPC who received tomotherapy using simultaneously integrated boost (SIB) strategies were replanned with VMAT (RapidArc of Varian, dual-arc). Dosimetric comparisons between the RapidArc plan and the HT plan included the following: (1) D98, homogeneity, and conformity of PTVs; (2) sparing of organs at risk (OARs); (3) delivery time and monitor units (MUs).

**Results:**

(1) Compared with RapidArc, HT achieved better dose conformity (CI of PGTVnx + nd: 0.861 versus 0.818,* P* = 0.004). (2) In terms of OAR protection, RapidArc exhibited significant superiority in sparing ipsilateral optic nerve (Dmax: 27.5Gy versus 49.1Gy,* P* < 0.001; D2: 23.5Gy versus 48.2Gy,* P* < 0.001), contralateral optic nerve (Dmax: 30.4Gy versus 49.2Gy,* P* < 0.001; D2: 26.2Gy versus 48.1Gy,* P* < 0.001), and optic chiasm (Dmax: 32.8Gy versus 48.3Gy,* P* < 0.001; D2: 30Gy versus 47.6Gy,* P* < 0.001). HT demonstrated a superior ability to protect the brain stem (D1cc: 43.0Gy versus 45.2Gy,* P* = 0.012), ipsilateral temporal lobe (Dmax 64.5Gy versus 66.4 Gy,* P* = 0.015), contralateral temporal lobe (Dmax: 62.8Gy versus 65.1Gy,* P* = 0.001), ipsilateral lens (Dmax: 4.27Gy versus 5.24Gy,* P* = 0.009; D2: 4.00Gy versus 5.05Gy,* P* = 0.002; Dmean: 2.99Gy versus 4.31Gy,* P* < 0.001), contralateral lens (Dmax: 4.25Gy versus 5.09Gy,* P* = 0.047; D2: 3.91Gy versus 4.92Gy,* P* = 0.005; Dmean: 2.91Gy versus 4.18Gy,* P* < 0.001), ipsilateral parotid (Dmean: 36.4Gy versus 41.1Gy,* P* = 0.002; V30Gy: 54.8% versus 70.4%,* P* = 0.009), and contralateral parotid (Dmean: 33.4Gy versus 39.1Gy,* P* < 0.001; V30Gy: 48.2% versus 67.3%,* P* = 0.005). There were no statistically significant differences in spinal cord or pituitary protection between the RapidArc plan and the HT plan. (3) RapidArc achieved a much shorter delivery time (3.8 min versus 7.5 min,* P* < 0.001) and a lower MU (618MUs versus 5646MUs,* P* < 0.001).

**Conclusion:**

Our results show that RapidArc and HT are comparable in D98, dose homogeneity, and protection of the spinal cord and pituitary gland. RapidArc performs better in shortening delivery time, lowering MUs, and sparing the optic nerve and optic chiasm. HT is superior in dose conformity and protection of the brain stem, temporal lobe, lens, and parotid.

## 1. Introduction

Nasopharyngeal carcinoma (NPC) is a common type of head and neck cancer (HNC) found in Southeast Asia and North Africa [1, 2]. Due to its deep anatomical location and high sensitivity to radiation, radiotherapy has become the principal method of treating NPC. The challenge of using radiotherapy for NPC lies in improving tumor control while reducing the amount of radiation delivered to nearby normal structures. With the development of intensity modulated radiotherapy (IMRT), which can deliver highly focused radiation to tumor targets, this problem has been virtually eliminated [3–5]. However, the performance of conventional IMRT is not perfect, particularly in cases where the primary tumor is close to critical structures such as the spinal cord and brainstem [6, 7].

Volumetric modulated arc therapy (VMAT), a new technique based on conventional IMRT, is reported to outperform conventional IMRT by modulating gantry rotation speed, dose rate, and shapes of multileaf collimator (MLC) [8, 9]. Several studies in NPC and other HNCs have demonstrated that VMAT can achieve a shorter delivery time, lower MUs, superior or equivalent dose coverage, and better OAR protection compared with conventional IMRT treatment [10–16].

Helical tomotherapy (HT), another novel technique which combines rotational radiation delivery, fast-switching binary MLC, and movable couch, has also shown great promise in cancer treatment with regard to dosimetric advantages [17]. Several published reports have indicated that HT is significantly superior to conventional IMRT in dose conformity, dose homogeneity, and protection of OARs when treating NPC and other HNCs [11, 13, 16, 18–21].

Many studies have demonstrated that both VMAT and HT can outperform conventional IMRT; comparisons between VMAT and HT are needed to inform the choice of treatment methods. There have been several studies comparing VMAT and HT in other HNCs, indicating that VMAT is better in shortening delivering time and protecting thyroid glands and optic nerves while HT is superior in dose conformity and sparing of lens and brain stem [4, 22–24]. However, very few publications have focused on such comparisons in NPC. Importantly, because the primary tumor of NPC is surrounded by critical structures such as the brain stem and optic structures, T-stage is a very important factor in the planning of radiotherapy [25]. For this reason, in this study, we compared the dosimetric differences between VMAT and HT in early T-stage NPC patients.

## 2. Materials and Methods

### 2.1. Patients

Ten patients with early T-stage NPC (2 cases of T1 and 8 cases of T2, according to 8th AJCC staging system) treated with HT were enrolled in this study. Written informed consent was obtained from each subject. Patient characteristics are listed in Table 1.

### 2.2. Simulation, Delineation, and Dose Requirements

For simulation, all patients were immobilized using thermoplastic masks, which spanned from head to shoulder. Intravenous contrast-enhanced computed tomography (CT) using a 3 mm slice thickness was performed, and the result was imported into the treatment planning system. Magnetic resonance images were used to assist delineation.

The target volumes consisted of GTVnx, GTVnd, CTV1, and CTV2. GTVnx was defined as the primary tumor identified via imaging or physical examination, GTVnd was defined as positive metastatic lymph nodes, and CTV was defined as the grossly detectable tumor volume plus microscopic tumors. CTV1 and CTV2 referred to the high-risk region and the low-risk region, respectively. Planning target volumes (PTVs) of PGTVnx, PGTVnd, PTV1, and PTV2 were generated from each corresponding target volume with an additional 3-5 mm margin to account for setup error and organ movement. The OARs included the brain stem, spinal cord, pituitary, optic chiasm, optic nerves, lenses, temporal lobes, and parotid glands.

The prescription dose of each PTV was given as follows: PGTVnx 68.1 - 70.4Gy, PGTVnd 68.1 - 70.4Gy, PTV1 59.2 - 60.8Gy, and PTV2 50.4 - 51.8Gy. For each PTV, at least 95% of the volume should be covered by its corresponding prescription dose. Regarding the OARs, the maximum doses to brain stem, spinal cord, pituitary, optic chiasm, optic nerve, and lens were set as 54Gy, 45Gy, 54Gy, 54Gy, 54Gy, and 9Gy, respectively. Additionally, the relative volume receiving over 30Gy should be less than 50%, at least for one side of the parotid glands, and the relative volume receiving over 60Gy should be less than 5% for the temporal lobes [11, 13].

### 2.3. Planning Techniques

VMAT plan: RapidArc (Varian Medical Systems, Palo Alto, CA) was used to perform VMAT. The eclipse treatment planning system (Eclipse version 11.3, Varian Medical Systems, Palo Alto, CA) was adopted to generate the RapidArc plan. Dose calculation was performed using the analytical anisotropic algorithm. The collimator was rotated by 0–15° to minimize the tongue and groove effect. The devised parameters included 2 coplanar full arcs, gantry rotation speed of 4.8deg/s, and adjustable dose rate (maximum dose rate 600MU/min).

HT plan: The tomotherapy plan was generated with the TomoTherapy Planning Workstation (TomoHD version 2.0.7, Accuracy Inc., Sunnyvale, CA). Dose calculation was performed using the convolution/superposition algorithm. The planning parameters were set as follows: field width = 2.5 cm, pitch = 0.287, and modulation factor = 2.0 - 2.6.

### 2.4. Plan Comparison

Dosimetric comparisons were performed based on the Dose-Volume Histogram (DVH). Target coverage, dose homogeneity, and dose conformity were represented by D98, homogeneity index (HI), and conformity index (CI), respectively [11]. HI and CI were calculated using the following equations [11]:

(1) HI=D2−D98D50;CI=TVRITV×TVRIVRI.

D2, D50, and D98 refer to the maximum dose encompassing 2%, 50%, and 98% of PTV, respectively. TV_RI_ is the volume of PTV covered by the prescribed dose, TV is the volume of PTV, and V_RI_ is the volume of the body covered by the prescribed dose. A lower HI indicates better homogeneity and a higher CI denotes superior conformity.

The following parameters were used to evaluate the protection of OARs: Dmax (maximum point dose), D0.1cc (maximum dose encompassing 0.1cc of the structure), and D1cc (maximum dose encompassing 1cc of the structure) for the spinal cord and brain stem [7, 14, 26, 27]; Dmax and D2 for the optic chiasm and optic nerve [11, 13]; Dmax and V60Gy (the relative volume of the structure receiving over 60Gy) for the temporal lobe [6, 28]; Dmax, D2, and Dmean for the lens [11, 25]; and Dmean, Dmedian, and V30Gy (the relative volume of the structure receiving over 30Gy) for the parotid [13]; Dmax for the pituitary [6]; V15/20/30/40Gy for the whole body (the relative volume of the whole body in scanning scope receiving over V15/20/30/40Gy) [14].

The number of monitor units (MU) of each plan was recorded in the planning system. Expected delivery time was calculated based on 4.8deg/s gantry rotation speed for RapidArc and 866 MU/min dose rate for HT. For RapidArc, 20 seconds was added for the switch between the two arcs and 1 minute was added for positioning. For HT, 1 minute was added for positioning.

### 2.5. Statistical Analysis

The paired *t*-test was performed to test comparisons between RapidArc and HT. A 2-tailed P-value of < 0.05 was considered statistically significant. All analyses were performed using SPSS (version 21, IBM SPSS Statistics).

## 3. Results

### 3.1. PTV Coverage

**Figure 1:**
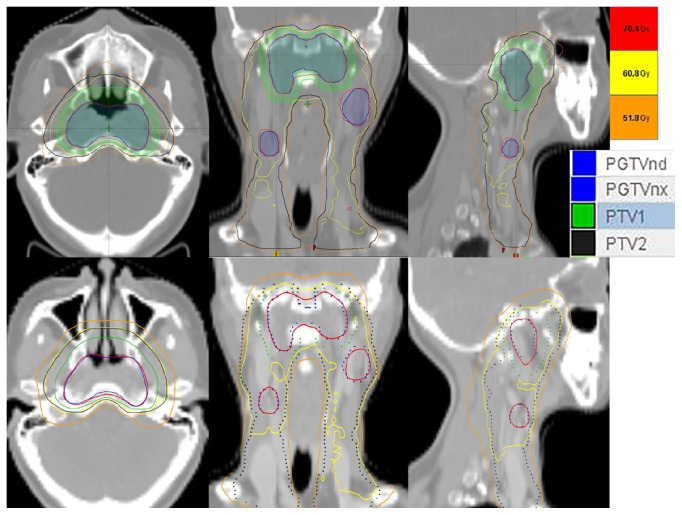
Dose distributions of a typical case planned by RapidArc (upper) and HT (lower). The red, yellow, and orange lines are isodose curves of 70.4Gy (prescription dose of PGTVnx/ PGTVnd), 60.8Gy (prescription dose of PTV1), and 51.8Gy (prescription dose of PTV2), respectively. The blue, green, and black lines represent the contours of PGTVnx/ PGTVnd, PTV1, and PTV2, respectively.

Figure 1 shows the dose distribution of a typical case planned by RapidArc and HT.  Table 2 shows the dosimetric parameter comparisons of PTVs between RapidArc and HT. RapidArc and HT achieved similar D98 and HI in all PTVs. However, HT showed better dose conformity than RapidArc in PGTVnx+nd (0.861 versus 0.818, P = 0.004).

### 3.2. OARs

Results of the statistical analysis for OAR protection are listed in Table 3. RapidArc showed significant superiority in sparing the ipsilateral optic nerve, contralateral optic nerve, and optic chiasm. Alternatively, HT exhibited an obvious advantage in protection of the brain stem, ipsilateral temporal lobe, contralateral temporal lobe, ipsilateral lens, contralateral lens, ipsilateral parotid, and contralateral parotid. There were no statistically significant differences in spinal cord and pituitary protection.

### 3.3. MUs and Delivery Time

As shown in Table 4, RapidArc had a significantly shorter delivery time (3.8 min versus 7.5 min, P < 0.001) and lower MU (618MUs versus 5646MUs, P < 0.001).

## 4. Discussion

VMAT and HT represent the most advanced photon radiotherapy techniques available. However, very few studies focused on dosimetric comparisons between VMAT and HT in NPC [11, 13]. Due to the proximity of the primary tumor and surrounding critical tissues, the location and size of the primary tumor are major factors in determining the complexity of the radiotherapy plan in NPC. This is supported by the study of Abbasi et al., which demonstrated that advanced T-stage, intracranial extension, and large tumor volume are important factors associated with decreased dose coverage of PTVs [25]. Therefore, consideration of the T-stage as an independent factor is more accurate when comparisons are performed. The current study is the first to perform dosimetric comparisons between VMAT and HT in early T-stage NPC.

In terms of PTV coverage, our study showed that HT is superior to RapidArc in dose conformity, although there was no difference in D98 and homogeneity between the two plans. This is consistent with several previous studies which have indicated that HT outperforms VMAT on dose conformity in NPC [11, 13] and other head and neck cancers [26, 29]. However, Liu X et al. reported that VMAT was better than HT in dose conformity in nasal natural killer/T-cell lymphoma [22]. This inconsistency may has something to do with the difference in research objects and setting parameters of techniques. Since dose conformity represents the congruence between isodoses and tumor contours [30], better conformity indicates potential superior tumor target coverage and OAR protection.

The VMAT and HT plans both demonstrated positive and negative properties in OAR protection. RapidArc exhibited an obvious advantage in sparing the optic nerve and chiasm. In the ipsilateral optic nerve, contralateral optic nerve, and optic chiasm, RapidArc reduced Dmax by 44%, 38%, and 32% and D2 by 51%, 45%, and 37%, respectively, compared with HT. This is in concordance with the studies of Lee et al. and Lu et al. [11, 13], which indicated that protection of the optic nerves and chiasm is a significant weakness of HT when compared with VMAT. The 2.5 cm field width and fixed jaw which can lead to craniocaudal dose spread are believed to be responsible for this phenomenon. With the recently developed dynamic jaw technology, which allows for smaller jaws at the cranial and caudal parts, there is an expectation that this problem is solved [31, 32].

In contrast to the advantages of RapidArc in optic nerve and chiasm protection, HT showed superiority in sparing of lenses, parotids, brainstem, and temporal lobes. In the ipsilateral lens, the Dmax, D2, and Dmean of the HT plan were 19%, 21%, and 31% lower than those of the RapidArc plan. In the contralateral lens, there was also a 17%, 21%, and 30% reduction of Dmax, D2, and Dmean, respectively, in the HT plan. This result is consistent with the study by Lee et al. [11], and the advantage may be attributed to the lower radiation transmission rate of binary MLC in tomotherapy [11]. Although both plans can satisfy the dose constraints of the lens, it is more beneficial to utilize the lowest lens dose possible, as recent studies have indicated that the dose threshold of the lens may be lower than previously suggested [33].

This study also demonstrated that for the ipsilateral and contralateral parotids, HT reduced Dmean by 12% and 15%, respectively, and V30Gy by 22% and 28%, respectively, compared with RapidArc. However, this finding is not consistent with the results of Lee et al. [11] and D Leignel et al. [34], which reported no significant difference in the Dmean of parotids between RapidArc and HT. The observed inconsistency may be due to the difference in research objects. Lee's research focused on late stage NPC; Leignel's study included oropharynx, oral cavity, and nasopharynx tumors, and the current study was focused on early T-stage NPC. Because xerostomia is a common and painful complication for NPC patients who receive radiotherapy, parotid sparing is an important factor in the evaluation of a treatment plan. Therefore, further studies are needed to assess the performance of VMAT and HT in protecting the parotids.

HT exhibited a 2.2Gy reduction in D1cc of the brainstem, although there were no differences in Dmax and D0.1cc between the two plans. Further, HT also demonstrated a 2.0Gy and 2.3Gy decrease in Dmax of the ipsilateral and contralateral temporal lobes, respectively, despite their similar performance in V60Gy. The advantage of HT in central nervous system protection is supported by several previous studies on other HNCs [23, 24]. In consideration of the incapacitating and devastating results of radiation-induced brainstem injury and temporal lobe necrosis, every possible measure should be taken to lower the dose to the central nervous system [18, 35, 36].

When considering MU and expected delivery time, RapidArc also showed an obvious advantage over HT. This study demonstrated that the delivery time of RapidArc was 3.7 minutes shorter than HT, and the MUs of RapidArc were approximately one-ninth of HT, which is consistent with previous studies [11, 13, 22, 29, 37]. Shorter delivery time is an advantage for patients who cannot tolerate a long treatment time and increases the usage rate of the machine. Lower MUs indicate a lower radiation dose through MLC [38], which was observed via the reduced V15Gy, V20Gy, V30Gy, and V40Gy of RapidArc, although the reduction was not statistically significant. Besides, it is worth mentioning that HT is generally more expensive than RapidArc [39].

The results of this study were potentially affected by several factors. First, the performance of VMAT may be influenced by the manufacturer and/or the arc numbers used in the treatment. The results of this study were based on the dual-arc RapidArc from Varian Medical Systems. In addition, due to current technique updates in the RapidArc and HT, such as the dynamic lead-jaw, performance may be improved. Second, because previous studies [7, 25] and clinical experience indicate that NPC T-stage is a major factor in determining the complexity of the radiotherapy plan, N-stage was not taken into consideration in this study. Further study using subgroup analysis on N-stage may be needed to determine more accurate results. Third, the sample size of this study is relatively small, which may affect the results. Finally, the setting parameters adopted in each technique were based on literature review and treatment experience in our hospital, which should also be taken into consideration when results are interpreted.

## 5. Conclusion

For early T-stage NPC, RapidArc and HT are comparable in D98, dose homogeneity, and protection of the spinal cord and pituitary. RapidArc performs better in shortening delivery time, lowering MUs, and sparing the optic nerve and optic chiasm; HT is superior in dose conformity and protection of the brain stem, temporal lobe, lens, and parotid.

## Figures and Tables

**Table 1:** Selected patients and tumor characteristics.

Case no.	Age	Sex	T stage	N stage	M stage	Clinical stage
01	40	M	2	2	0	III
02	52	F	1	3	0	IV
03	53	M	2	2	0	III
04	38	F	2	0	0	II
05	64	M	2	3	0	IV
06	52	F	2	0	0	II
07	42	M	2	1	0	II
08	53	M	2	1	0	II
09	53	M	1	2	0	III
10	41	M	2	1	0	II

Tumor stage is based on the 8th AJCC staging system. PGTVnx, PGTVnd, PTV1, and PTV2 refer to the PTV of primary tumor of nasopharynx, positive metastatic lymph nodes, high risk region, and low risk region, respectively.

**Table 2:** Dosimetric comparisons of PTVs between RapidArc and HT plans.

		RapidArc	HT	P-value
		Average (SD)	Average (SD)	
D98(cGy)	PGTVnx	6930 (91)	6938 (88)	NS
	PGTVnd	6978 (83)	6964 (102)	NS
	PTV1	6066 (154)	6098 (146)	NS
	PTV2	5331 (258)	5391 (144)	NS
HI	PGTVnx	0.073 (0.013)	0.073 (0.013)	NS
	PGTVnd	0.076 (0.011)	0.07 (0.011)	NS
	PTV1	0.201 (0.034)	0.205 (0.026)	NS
	PTV2	0.31 (0.071)	0.297 (0.073)	NS
CI	PGTVnx+nd	0.818 (0.025)	0.861 (0.033)	0.004
	PTV1	0.453 (0.246)	0.515 (0.268)	NS
	PTV2	0.627 (0.269)	0.591 (0.245)	NS

SD: standard deviation; D98: the maximum dose encompassing 98% of PTV; HI: homogeneity index; CI: conformity index. The CI of PGTVnx and PGTVnd were calculated integrally because they were given the same prescription dose.

**Table 3:** Dosimetric comparisons of OARs between RapidArc and HT plans.

OAR	Dose Parameter	RapidArc		HT		P-value
		Average (SD)		Average (SD)		
Spinal Cord	Dmax (cGy)	3603	(368)	3412	(149)	NS
	D1cc (cGy)	3331	(400)	3240	(131)	NS
	D0.1cc (cGy)	3429	(408)	3347	(132)	NS
Brain Stem	Dmax (cGy)	4965	(130)	4912	(103)	NS
	D1cc (cGy)	4524	(146)	4304	(179)	0.012
	D0.1cc (cGy)	4767	(149)	4684	(151)	NS
Optic Chiasm	Dmax (cGy)	3282	(1436)	4829	(735)	<0.001
	D2 (cGy)	3003	(1220)	4763	(736)	<0.001
Optic Nerve I	Dmax (cGy)	2745	(1728)	4906	(1025)	<0.001
	D2 (cGy)	2352	(1578)	4822	(989)	<0.001
Optic Nerve C	Dmax (cGy)	3040	(1595)	4921	(763)	<0.001
	D2 (cGy)	2621	(1419)	4807	(731)	<0.001
Temporal Lobe I	Dmax (cGy)	6643	(350)	6447	(285)	0.015
	V60Gy (%)	1.7	(1.3)	2.1	(1.6)	NS
Temporal Lobe C	Dmax (cGy)	6512	(101)	6283	(162)	0.001
	V60Gy (%)	1	(0.5)	1.1	(0.6)	NS
Lens I	Dmax (cGy)	524	(98)	427	(119)	0.009
	D2 (cGy)	505	(93)	400	(94)	0.002
	Dmean (cGy)	431	(97)	299	(46)	<0.001
Lens C	Dmax (cGy)	509	(87)	425	(135)	0.047
	D2 (cGy)	492	(86)	391	(100)	0.005
	Dmean (cGy)	418	(92)	291	(47)	<0.001
Parotid I	Dmean (cGy)	4114	(493)	3636	(514)	0.002
	Dmedian (cGy)	3791	(618)	3437	(809)	NS
	V30Gy (%)	70.4	(13.9)	54.8	(9.8)	0.009
Parotid C	Dmean (cGy)	3909	(431)	3336	(449)	<0.001
	Dmedian (cGy)	3585	(525)	3033	(658)	0.018
	V30Gy (%)	67.3	(12.7)	48.2	(9.6)	0.005
Pituitary	Dmax (cGy)	5548	(1023)	5786	(521)	NS
Body	V15 Gy (%)	32.1	(5.2)	40	(15.1)	NS
	V20 Gy (%)	27.9	(4.9)	34.5	(13.7)	NS
	V30 Gy (%)	19.9	(4.3)	23.1	(11)	NS
	V40 Gy (%)	12.5	(2.9)	14.2	(6.7)	NS

OAR: organ at risk; SD: standard deviation; Dmax: maximum point dose; D1cc: maximum dose encompassing 1cc of the structure; D0.1cc: maximum dose encompassing 0.1cc of the structure; D2: maximum dose encompassing 2% of the structure; Dmean: mean dose of the structure; Dmedian: median dose of the structure; V15,20,30,40,60 Gy: the relative volume of the structure receiving over 15,20,30,40,60Gy.

**Table 4:** MUs and expected delivery time comparisons between RapidArc and HT plans.

	RapidArc	Tomotherapy	P-value
	Average (SD)	Average (SD)	
MUs	618.2 (82.9)	5646.32 (642.6)	<0.001
Expected Delivery time (min)	3.8 (0)	7.5 (0.7)	<0.001

MUs: monitor units
